# An Overview of Parenteral Nutrition from Birth to Adolescence Based on a Composite Fish Oil Containing Lipid Emulsion and a Pediatric Amino Acid Solution

**DOI:** 10.3390/nu16030440

**Published:** 2024-02-01

**Authors:** Olivier Goulet

**Affiliations:** Faculté de Médecine Paris Centre, Descartes Medical School, Université de Paris, 75006 Paris, France; olivier.goulet-ext@aphp.fr

**Keywords:** intestinal failure, parenteral nutrition, home parenteral nutrition, fish-oil-based intravenous lipid emulsion, crystalline amino acids solution, multi-chamber bags

## Abstract

Intestinal failure (IF) is characterized by a critical reduction in functional gut mass below the minimum needed for optimal growth in children. It requires parenteral nutrition (PN) and home-PN (HPN), which is challenging in terms of meeting nutritional needs according to age, growth velocity, clinical situation, and rapid changes in fluid and electrolyte requirements. Due to these complex requirements, age-adapted multi-chamber bags (MCBs) are important additions to the nutrition armamentarium. The launch of composite fish oil (FO)-containing intravenous lipid emulsions (ILEs) heralded the development of MCBs containing these ILEs in combination with a crystalline amino acid solution adapted for pediatric use. The safety and efficacy of lipid and amino acid components in this context have been widely documented in numerous published studies. This narrative manuscript includes a review of the articles published in PudMed, Embase, and Google Scholar up to June 2023 for the age groups of term infants to children and adolescents. Preterm infants with their highly specific demands are not included. It aims to offer an overview of the clinical experience regarding the use of a composite FO-based ILE and a developed specific amino acid solution.

## 1. Introduction

Adequate nutrient supply is of greater relevance during childhood than at any other period in life. Due to their high basal and anabolic requirements and limited metabolic reserves, pediatric patients are particularly sensitive to energy and protein restriction [1]. Sufficient nutrients must be provided not only for the maintenance of body tissue, but also for growth. Starvation, even for few days, may be detrimental [1,2,3].

Parenteral nutrition (PN) has become a valid therapeutic option for patients with intestinal failure (IF) who require long-term PN and home-PN (HPN) [1,4,5]. The provision of PN to pediatric patients represents a distinct clinical challenge, as metabolic demands and nutritional needs change according to age, growth velocity, and clinical situations [3]. Moreover, complex and rapidly changing fluid and electrolyte requirements are a major challenge to PN delivery [6].

In children, IF is defined as a critical reduction in functional gut mass below the minimum needed for adequate growth [7,8]. The leading cause of IF in childhood is short bowel syndrome as a consequence of extensive small bowel resection. Long-term PN and home-PN (HPN) are the mainstay for the management of chronic IF, and allow children to grow in their familiar environment [9].

This narrative review aims to give an overview of specific quantitative and qualitative nutritional intake in pediatric patients including term newborn infants, infants/toddlers, children, and adolescents receiving PN. Preterm infants with their highly specific demands are not included, as this would be beyond the scope of the present article. Moreover, it explores the clinical experience regarding the use of a composite intravenous lipid emulsion (ILE) containing fish oil (FO) (SMOFlipid^®^, Fresenius Kabi, Bad Homburg, Germany) and a pediatric crystalline amino acid solution (Vaminolact^®^, Fresenius Kabi, Bad Homburg, Germany) in the management of pediatric patients with acute or chronic IF depending on PN.

To this end, we conducted a literature search sourcing published research investigating the use of SMOFlipid and Vaminolact in the management of pediatric patients with acute or chronic IF depending on PN. Databases searched were PubMed (without any time restrictions), Embase (papers published since 2016), and Google Scholar (papers published since 2018), with the last search conducted in June 2022, and an update in January 2024. Retrospective studies, prospective studies, and RCTs conducted on the age groups of newborn infants, infants/toddlers, children, or adolescents and reporting efficacy and/or safety results were eligible for this narrative review. Studies conducted exclusively on preterm newborns, studies on adults, abstracts, case reports, studies not investigating PN effects/the off-label use of SMOFlipid or Vaminolact, and studies with unclear data regarding the actual use of SMOFlipid/Vaminolact were excluded.

## 2. Provision of Energy and Macronutrients in Pediatric Patients on PN

### 2.1. Energy

Generally, energy supply should meet the requirements to cover resting energy expenditure, plus those for physical activity, growth, diet-induced thermogenesis, and the correction of pre-existing malnutrition when present [3,10]. ESPGHAN/ESPEN/ESPR guidelines recommend calculating total parenteral energy requirements of stable patients from the resting energy expenditure, which may be either measured by indirect calorimetry or estimated from Schofield’s formula based on gender, age, body weight, and length [11]. Energy intake must cover energy expenditure for physical activity and growth (including catch-up growth) and must be adjusted according to age and disease states [3].

In clinical practice, PN intake must be adapted to the degree of intestinal insufficiency in pediatric patients. It may be estimated by the level of PN required for normal or catch-up growth. PN dependency index (PNDI) is defined as the ratio of non-protein-energy intake (NPEI) provided by PN for achieving optimal growth to the resting energy expenditure (REE). REE is calculated by using the Schofield formulas. The PNDI has been validated from several papers [12,13,14,15]. In clinical practice, the PNDI corresponds to the caloric requirements from PN to achieve normal growth. It reflects the degree of intestinal insufficiency more accurately than other criteria, such as the ratio of parenteral versus enteral intakes, or the addition of PN and enteral caloric intake when compared to recommended dietary allowances. The PNDI for estimating the PN requirements for optimal growth considers the final nutritional efficiency. Based on the PNDI expressed as a percentage, it can be considered high when >120%, and even higher for achieving catch-up growth, and low when <50% [12,13,14,15].

Excess energy intake may increase the risk of hyperglycemia, infections, and fat deposition (fat body mass and liver steatosis). Underfeeding may result in failure to thrive, poor development, and impaired immune responses, increasing the risk of morbidity and mortality in newborn infants, infants/toddlers, and children [3]. In acute critical illness, energy provision should be reduced compared to stable patients, while overfeeding (glucose) increases ventilatory workload and potentially prolongs the need for mechanical ventilation and the length of hospital stay [3,16]. During the stable phase of critical illness, requirements are approximately 1.3 times the resting energy expenditure and further increase in the recovery phase for catch-up growth [3]. Therefore, energy provision must be carefully managed to ensure that energy needs are met but not exceeded.

### 2.2. Glucose

D-glucose (dextrose) is the main source of calories in PN, but also accounts for a major part of the osmolarity in PN solutions. All body cells and organs utilize glucose, and for the brain, renal medulla, and erythrocytes, glucose is an obligatory energy substrate [17].

Factors guiding glucose intake with PN include age, disease status, nutritional status, and the concomitant provision of other macronutrients (lipids and amino acids). The rate of glucose delivery should not exceed the maximum rate of glucose oxidation. Studies have shown a maximal oxidation rate of about 12 mg/kg body weight (BW) per min (17.2 g/kg BW per day) in term infants after surgery or infants on long-term PN [17,18,19].

The higher the glucose delivery rate, the higher the rate of hyperinsulinism, favoring a costly lipid synthesis from glucose (lipogenesis) with subsequent lipid deposition in adipose tissue and liver. Moreover, glucose overfeeding induces hyperglycemia and increases CO_2_ production and minute ventilation [17]. In critically ill children with insulin resistance and beta-cell dysfunction, risk of hyperglycemia is a particular concern [17,20] and represents an independent risk factor for morbidity and mortality [21,22].

### 2.3. Lipids

Intravenous lipid emulsions are an integral component of pediatric PN [23]. ILEs are a low-osmolarity and energy-dense source of calories. In addition, they serve as a source of essential fatty acids (EFAs) and facilitate the delivery of fat-soluble vitamins [24,25]. According to the ESPGHAN/ESPEN/ESPR guidelines [23], lipid intake should provide 25–50% of non-protein calories in pediatric patients on total PN. As for glucose, the maximal lipid oxidation rate has been established [26]. It decreases with age, from approximately 3 g/kg/day in young children to 1.7–2.5 g/kg/day in adults. According to ESPGHAN/ESPEN/ESPR guidelines [23], parenteral lipid intake in children should be limited to a maximum of 3 g/kg/day.

To prevent biochemical evidence of essential fatty acid (EFA) deficiency, parenteral lipid intake should include 0.1 g/kg BW/day of omega-6 polyunsaturated fatty acid (PUFA) linoleic acid in term infants and children [23]. “Historical” ILEs, subsequently referred to as “first generation”, were derived from pure soybean oil (SO) rich in LA. A potential disadvantage with pure SO ILEs is that high contents of omega-6 PUFAs, in particular of LA, have been attributed to pro-inflammatory and immunosuppressive properties [27]. This led to the development of more advanced ILEs containing mixtures of SO with alternative oil sources such as medium-chain triglycerides (MCTs) from coconut/palm kernel oil, olive oil (OO), and/or FO [25,28]. In particular, FO, representing a source of omega-3 PUFAs with anti-inflammatory properties [29], has become an important component of the last generation of composite ILEs containing FO [27,28]. Long-chain omega-3 PUFA docosahexaenoic acid (DHA), eicosapentaenoic acid (EPA), and omega-6 PUFA arachidonic acid (ARA) modulate key metabolic pathways, including inflammatory and immune responses, coagulation, and cell signaling [28,30]. DHA and ARA are also required for an optimal development of the brain, as well as for cognition and visual acuity [31,32,33]. In order to prevent EFA deficiency in term infants and children, a lipid emulsion dosage providing a minimum linoleic acid intake of 0.1 g/kg/day can be given, which also provides an adequate intake of linolenic acid (LNA) with all 20% ILEs currently registered for pediatric use. In critically ill pediatric patients, ILE should also be an integral part of PN. Composite ILEs containing FO may be used as the first-choice treatment. Available evidence raises the important question on the best timing to provide PN support in critically ill children, but does not allow us to differentiate potential effects on outcomes of the timing of introducing ILE [21].

The ESPGHAN/ESPEN/ESPR guidelines support that, in newborn infants and older children on short-term PN, pure SO ILEs may provide a less balanced FA profile than composite ILEs [23]. For PN lasting longer than a few days, the use of pure SO-ILE is discouraged and composite ILEs with or without FO are recommended as first-line treatment [23,34]. Of note, to date there are no quantitative recommendations for the supply of EPA, DHA, or ARA in pediatric patients.

Intestinal-failure-associated liver disease (IFALD) is the most prevalent complication affecting children with IF receiving long-term PN [24,35,36]. The diagnosis of IFALD is usually based on the onset of cholestasis, generally defined as an elevation in conjugated serum bilirubin (CBil) concentration (≥2 mg/dL) [24,36]. Effects of pure FO-based ILEs (Omegaven^®^, Fresenius Kabi, Bad Homburg, Germany) for reversing cholestasis in children receiving an SO-based ILE (Intralipid^®^, Fresenius Kabi, Bad Homburg, Germany) were first reported by Gura et al. [37]. Ten years later, the same group reported data on patients treated with pure FO from 2004 to 2014. Most of the time, cholestasis appeared very early during the first year of life. Resolution of cholestasis was defined as sustained direct bilirubin (DBil) < 2 mg/dL, and treatment failure as liver transplantation or death. Among 182 patients treated with pure FO-ILE, 86% achieved resolution of cholestasis and 14% failed therapy. Factors significantly associated with therapy failure included lower birth weight, patients older than 20.4 weeks (9.9, 38.6 weeks) compared with 11.7 weeks (7.3, 21.4 weeks), and more advanced liver disease at therapy initiation compared to patients whose cholestasis resolved [38].

The provision of high amounts of omega-6 PUFA LA with pure SO ILEs results in the endogenous formation of ARA, a precursor of lipid mediators with more pro-inflammatory properties [28,39]. The excessive intake of linoleic acid may thus promote an inflammatory state contributing to cholestasis and liver fibrosis [30,39]. Moreover, pure SO ILEs are relatively low in vitamin E (alpha-tocopherol) as an antioxidant substrate and, thus, are prone to the formation of lipid peroxidation products, which may lead to macrophage activation and hepatocyte damage [24,40,41]. Finally, pure SO-based ILEs also contain much higher concentrations of phytosterols compared with any of other ILEs, especially composite ILEs containing FO [42]. There is evidence that phytosterols may accumulate in the liver, thereby reducing bile secretion while promoting liver damage in children with IF [24,43]. The recent discovery of farnesoid X receptor (FXR) as a possible target mediating altered bile secretion in short bowel syndrome patients has opened a new field of research for IFALD prevention [44,45,46].

In this regard, composite ILEs with FO offer several advantages, including high concentrations of anti-inflammatory omega-3 PUFAs, DHA, and EPA, the antioxidant alpha-tocopherol (200 mg/L), reduced omega-6 PUFA content, and a reduced phytosterol load [23,24,39]. Moreover, some composite ILEs containing FO also provide rapidly oxidizable MCTs [47,48], while the diversity of oils ensures a balanced intake of FA and prevents EFA deficiency [35,36].

To reverse IFALD in pediatric patients, ESPGHAN recommends a discontinuation of SO ILE, a reduction in other ILE dosages, and/or the use of composite ILEs with FO, along with the treatment and management of other risks. The use of pure FO ILE is not recommended for general use in pediatric patients but may be used for short-term rescue treatment in patients with progression to severe IFALD, based on the case.

### 2.4. Amino Acids

In any patient receiving PN, proteins have to be administered in the form of free amino acids. Pediatric amino acid solutions are currently available. Their primary goal is to promote anabolism (protein synthesis and positive nitrogen balance) for cells and tissue building (e.g., muscle, skeleton) consistent with the normal growth and development typical of healthy peers [49]. Due to additional needs for growth, the protein requirements based on weight are higher in newborn infants, infants/toddlers, and children than in adults, and the timely provision of adequate amounts of amino acids with PN is particularly critical [1].

Generally, the total amino acid requirement is lower in parenterally fed newborn infants, infants/toddlers, and children than in enterally fed patients because the intestinal and hepatic “first-pass” metabolism is bypassed [50]. The ESPGHAN/ESPEN/ESPR guidelines [50] recommend a minimum parenteral amino acid intake of 1.0 g/kg/d for stable infants and children from 1 month to 3 years to avoid negative balance (LOE 1¡, moderate quality, RG B, strong recommendation, strong consensus). In stable children aged 3–12 years, an amino acid intake of 1.0–2.0 g/kg per day may be considered (LOE 4, RG GPP, conditional recommendation, strong consensus) [48]. The maximum amino acid intake should not exceed 3.0 g/kg BW/day in term newborn infants and 2.0 g/kg BW/day in stable children and adolescents [50]. Excessive amino acid intake has been shown to increase oxidation, in turn promoting high blood urea concentrations and metabolic acidosis [51]. Of note, amino acids must always be co-administered with sufficient amounts of energy in the form of glucose and lipid to prevent the wasting of amino acids for endogenous glucose production. The optimal ratio of NPEI in kcal to 1 gr of nitrogen (N) decreases with age, from approximately 250:1 in infants below 1 year of age to 150:1 in adolescents [13].

In the 1960s, in the early phase of PN development, newborn infants received protein hydrolysates as a source of nitrogen [52,53]. These protein hydrolysates were not only nutritionally unadapted, but raised the risk of severe allergic reactions and were therefore abandoned. With the advent of technology for the production of pure L-amino acids, a second generation of amino acid solution was developed, but was much more adapted for adult than pediatric patients, and especially VLBW newborn infants. Extrapolation from data on oral nutrition was often unsatisfactory for the adequate formulation of such mixtures. The intestinal absorption metabolism of specific amino acids such as branched-chain amino acids (BCAAs), arginine, tyrosine, and methionine [54,55,56] is highly variable and may also change with age [50]. Accordingly, the composition of amino acid solutions for pediatric use should ideally be adapted to the specific requirements resulting from the lack of the intestinal “first-pass” metabolism. In other words, the composition of amino acid solutions for PN cannot copy the enteral intake.

In the 1980s, great attention was given to amino acid supply in very-low-birth-weight infants from the first day of life in order to avoid catabolism, establish anabolism, achieve in utero protein accretion rates, and promote linear growth. Numerous studies have been performed for developing amino acid solutions with the aim of resembling the plasma amino acid patterns of normally growing, breast-fed infants, or resembling the amino acid profile of cord blood and achieving positive nitrogen balance. The direct study of parenterally nourished patients led to the third generation of amino acid solutions, providing all nine essential amino acids and a varying composition of non-essential amino acids. Studies have been published on the clinical efficacy of altered doses of arginine, branched-chain amino acids, cysteine, and taurine supplementation in PN [57,58,59,60,61,62,63,64,65,66].

Today, the most currently used amino acid mixtures contain amino acid amounts that result from this model. These pediatric amino acid mixtures provide more essential and less non-essential amino acids. Specificities of these mixtures such as Vaminolact or Primene^®^ (Baxter Maurepas, France) are nowadays well established.

An amino acid that should be included in pediatric amino acid solutions is taurine. Plasma, platelet, and urinary taurine levels have been shown to be significantly decreased in children on long-term HPN compared to those in normal children of a similar age [67]. It has even been suggested that taurine may be conditionally essential in pediatric patients on long-term PN [68]. ESPGHAN/ESPEN/ESPR guidelines [50] recommend that amino acid solutions for infants and children should contain taurine, a recommendation that is based on data indicating that adequate taurine may prevent cholestasis in newborn infants.

Factors affecting solubilities of calcium and phosphate in neonatal PN solutions containing the new amino acid formulation were examined [69,70]. Nowadays, these “pediatric amino acid mixtures” are widely used for children and adolescents on short- as well as for long-term PN.

## 3. PN with a Composite Fish Oil Containing Lipid Emulsion in Newborn Infants, Infants/Toddlers, Children, and Adolescents—Clinical Experience

**Figure 1:**
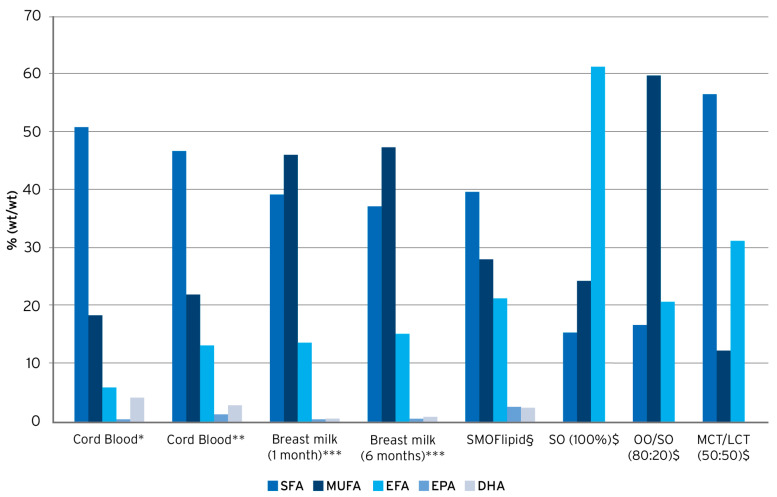
Fatty acid pattern of lipid emulsions vs. human umbilical cord blood and human breast milk. Compiled from * Agostoni, 2011 [74], ** Oliveira et al., 2012 [73], *** Koletzko et al., 2016 [75], § Goulet et al., 2010 [48], and $ Koletzko et al., 2005 [2].

SMOFlipid is a composite ILE containing SO (30%), MCTs (30%), OO (25%), and FO (15%), and is supplemented with alpha-tocopherol (200 mg/L) to counteract lipid peroxidation and oxidative stress [22]. A key feature of SMOFlipid is its specific fatty acid pattern resulting from the combination of these four different oils. In comparison to SO, SO/MCT, or OO/SO-based ILEs, the pattern is more similar to human umbilical cord blood and breast milk, respectively, with regard to proportions of saturated fatty acids, monounsaturated fatty acids, EFAs, and the omega-3 PUFAs EPA and DHA (Figure 1) [71,72,73]. Such a balanced fatty acid supply is of relevance, especially for newborn infants, to maintain plasma and tissue fatty acid patterns equivalent to those seen in breast-fed infants [31].

Several randomized clinical trials have been performed in very-low-birth-weight infants comparing SO-based ILEs and SMOFlipid [76,77,78,79,80,81]. In these studies, growth was not different between groups, but those who received SMOFlipid had higher circulating EPA and DHA levels in both plasma and red blood cells (RBCs). Moreover, liver function tests (ALAT, ASAT, GGT, alkaline phosphatase) are less modified in those receiving FO-based ILEs. In numerous clinical trials conducted on term newborn infants, infants and toddlers, older children, and adolescents, SMOFlipid provided as a component of PN has been evaluated in comparison to other types of ILEs, especially SO-based ILEs, in terms of effects on plasma and tissue fatty acid patterns, inflammation and infections, cholestasis and liver function, growth and nutritional status, as well as EFA status (see Table 1).

### 3.1. Effects on Fatty Acid Patterns

Several studies evaluated the effects of HPN with SMOFlipid on fatty acid patterns in pediatric patients (Table 1). Goulet et al. conducted the first double-blind RCT including infants and children on HPN receiving either SMOFlipid or an SO-based ILE [48]. Both ILEs were administered at a mean dose of 1.4 g lipids/kg BW/day. After 4 weeks, the EPA and DHA contents of RBC and plasma phospholipids (PL) were significantly increased in the SMOFlipid group compared to the controls. Moreover, the ratio of n-3 to n-6 FAs in plasma and RBC-PL was significantly elevated with SMOFlipid compared to SO-ILE [48]. Similar results have been published by Lezo et al. [92] (Table 1) from a study involving 38 pediatric patients with a median age of 5.56 (0.9–21.9) years on long-term PN receiving composite FO-containing ILE (SMOFlipid) (n = 23) or OO-based ILE (Clinoleic) (n = 15) at a dose of 1.3 g lipids/kg BW/day.

It has been claimed that pure FO-based ILEs (e.g., Omegaven) are safe for achieving a normal EFA status [113]. Yet, ESPGHAN does not recommend the long-term use of Omegaven as the sole source of IV lipid [23]. As a corollary, the effects of the long-term use of SMOFlipid for children on long-term HPN receiving a composite FO-based ILE on RBC fatty acid profile and EFA status also remain to be clarified. With that aim, 46 children highly dependent on PN (median PNDI 120% and daily ILE dose of 1.5–2 g/kg BW) for ≥1 year were included in a prospective study when they had received the composite FO ILE for >6 month. Out of this baseline group, only 25 children remained highly PN-dependent (SMOF1, n = 25) and could be assessed a second time, 2.4 years later (SMOF2, n = 25). An independent control group (“weaned off PN” group; n = 24) included children with short bowel syndrome who received SMOFlipid and were weaned off PN for >2 years (median: 4 years). No differences for growth parameters, citrulline, or bilirubin were observed between the SMOF groups (0.2 < *p* < 0.8). The weaned-off group did not differ from the SMOF groups for growth parameters (0.2 < *p* < 0.4), but citrulline was higher (*p* < 0.0001), reflecting intestinal sufficiency. SMOFlipid induced higher percentages of EPA (8.4% ± 2.9%) and DHA (11.7% ± 2.2%) in RBC profiles in the SMOF2 group compared to weaned-off children (0.8% ± 0.4% and 6.6% ± 2.3%, respectively), but lower proportions of ARA. However, the Holman index, a marker of EFA deficiency, did not vary between groups (*p* = 0.9). Authors concluded that the long-term (>2.4 years) use of SMOFlipid was well tolerated in HPN-dependent children. The RBC fatty acid profile alterations were consistent with the omega-3 PUFA-enriched composition of this emulsion without evidence of EFA deficiency (see Table 1) [15].

Likewise, Lezo et al. found no significant differences in the Holman index between pediatric patients of all age groups receiving HPN with SMOFlipid or OO/SO-ILE (Clinoleic) compared to the healthy controls [92].

### 3.2. Effects on Inflammation and Infection

Evidence on the effects of PN with SMOFlipid on the parameters of inflammation and infection in hospitalized patients is limited and lacking for patients on HPN. In a small RCT measuring levels of pro-inflammatory cytokines in 14 newborn infants receiving SMOFlipid (dose range 1–4 g lipids/kg BW/day) for 72 h post gastrointestinal (GI) surgery, serum levels of IL-6 decreased from baseline to day 3, whereas there was an increase in IL-6 levels in controls receiving SO/MCT-ILE (*p* = 0.007) [96]. The authors concluded that SMOFlipid was beneficial to ameliorate the inflammatory response post GI-surgery [96]. In a larger RCT including 160 newborn infants receiving either SMOFlipid or SO/MCT-ILE for 4–5 days after GI surgery (lipid dose 1–3 g/kg BW/day), no significant differences in pro-inflammatory cytokines or incidence of sepsis between groups were seen at 2- and 4-weeks post-surgery [100].

### 3.3. Effects on Cholestasis/Liver Function

In hospitalized newborn infants, infants/toddlers, children, and/or adolescents receiving PN with SMOFlipid administered at doses of up to 3 g lipids/kg BW/day for periods of 1 to 9 weeks, studies report significantly lower incidences of cholestasis and IFALD, as reflected by significantly lower plasma-conjugated bilirubin (CBil), total bilirubin (TBil), and direct bilirubin (DBil) levels compared to controls receiving SO-ILE [88,91,98,99,101,104]. Moreover, the administration of SMOFlipid resulted in significantly lower levels of liver enzyme and DBil compared to SO/MCT-ILE [100,107,110].

In 2010, a double-blind RCT investigated the effects of SMOFlipid (lipid dose 1.4 g/kg BW/day), compared to the same dose of SO-ILE, on RBC fatty acid profiles and liver status in children receiving HPN over a period of 4 weeks. Total bilirubin decreased significantly with SMOFlipid, whereas it increased with SO-ILE over the observation period, with the change being significant between the treatment groups [48]. Muhammed et al. [112] published a cohort comparing serum bilirubin for a duration of 6 months in children with PN-associated jaundice who changed from SO-based ILE Intralipid (n = 9) to SMOFlipid (n = 8). After 6 months, 5 of 8 children in the SMOFlipid group and 2 of 9 children in the Intralipid group had total resolution of jaundice. The median bilirubin fell by 99 μmol/L in the SMOFlipid group but increased by 79 μmol/L in the Intralipid group (*p* = 0.02). In a retrospective study on infants, Belza et al. found that SMOFlipid administered at doses between 2 and 3 g lipid/kg BW/day resulted in a lower incidence and decreased severity of IFALD over the first 12 months of administration compared with a historical cohort who received SO-ILE. Infants receiving SMOFlipid were less likely to reach critical CBil levels, and no infant receiving SMOFlipid required “rescue treatment” with pure FO-ILE for the salvage of liver function [99]. In a large cross-sectional study including 578 pediatric patients enrolled in HPN centers in France between 2014 and 2019, it was reported that the use of SMOFlipid for these patients significantly increased within this period from 67.4% to 88.3%. Overall, the prevalence of cholestasis was low and remained stable between 4.1 and 5.9% during the study period [9]. In a recent prospective cross-sectional study including 46 children on HPN who were highly dependent upon PN, as indicated by the PNDI (median 120%), these investigators evaluated the clinical safety of SMOFlipid long-term administration (average duration 2.4 years) at doses between 1.5 and 2.0 g/kg BW/day [15]. Liver function tests (ALAT, ASAT, GGT, alkaline phosphatase) and indicators of cholestasis, especially CBil, were close to the normal range in the group receiving SMOFlipid. These results provide further evidence that PN with SMOFlipid reduces the risk of cholestasis and IFALD in the long term [15].

Overall, studies investigating effects of HPN with SMOFlipid (doses up to 3 g lipids/kg BW/day) for varying durations ranging from 4 weeks to 6.6 years consistently report benefits in terms of the correction or prevention of liver disease following SMOFlipid administration [48,90,94,112].

### 3.4. Effects on Growth and Nutritional Status

In studies evaluating the effects of PN with SMOFlipid, administered in doses up to 3 g lipids /kg BW/day for 2 to 9 weeks to hospitalized pediatric patients of all age groups, no significant differences in weight gain or parameters of growth as compared to SO-ILE [88,101,110] or SO/MCT-ILE [100,107] were reported (see Table 1), indicating that SMOFlipid is at least equally as effective as SO-ILE for supporting adequate growth.

Several studies evaluated the effects of long-term HPN with SMOFlipid on growth parameters (see Table 1). Goulet et al. found that after receiving SMOFlipid for more than 6 months, highly PN-dependent children (median age 5.8 years) on HPN showed normal body weight and height relative to growth charts for healthy children. After an extended PN duration of 2.4 years, growth parameters between patients still receiving PN with SMOFlipid and those weaned from PN did not show any significant differences and were consistent with normal growth rates [15]. Ho et al. retrospectively evaluated growth parameters in children on HPN from the initiation of SMOFlipid up to 1.5 years post SMOFlipid initiation and found slight increases in body mass index (BMI) z-scores over this period that were, however, not significant [94]. In newborn infants with IF receiving PN with SMOFlipid (2–3 g lipids/kg BW/day) for an average of 421 days, Belza et al. reported significantly improved weight z-scores after 3 and 6 months compared to historic controls receiving SO-ILE. Anthropometric parameters including weight, height, and head circumference remained within the normal range with both ILEs [99]. Lezo et al. conducted a prospective multicenter study including pediatric patients of all age groups receiving HPN with SMOFlipid or OO/SO-ILE (median lipid dose 1.3 g/kg BW/day) for a mean duration of 22 and 21 months, respectively [92]. Median Z-scores of weight, height, or BMI for age were not statistically significant between groups [92]. The authors concluded that both ILEs ensured adequate growth, despite the lower plasma levels of ARA seen in this study.

## 4. PN with a Pediatric Amino Acid Solution for Newborn Infants, Infants/Toddlers, Children, and Adolescents—Clinical Experience

**Figure 2:**
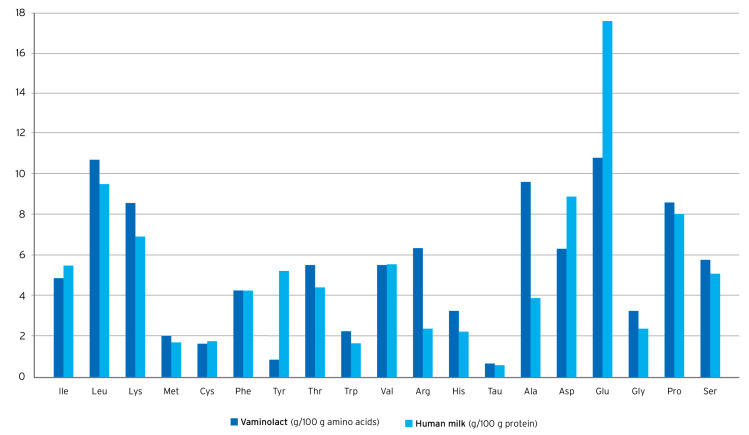
Amino acid supply to infants with human milk* or Vaminolact. *derived from WHO, 2007 [114], and Stapleton, 1997 [115].

Vaminolact is a pediatric amino acid solution providing essential (49%) and non-essential (51%) amino acids in a pattern similar to human milk (Figure 2). The amino acid composition of Vaminolact has a special focus on amino acids that are critical for pediatric patients (Table 2). It supplies adequate amounts of cysteine and tyrosine to compensate for the insufficient synthetic capacity in newborn and young infants. Moreover, it provides higher amounts of arginine to account for reduced intestinal synthesis and lower amounts of methionine, phenylalanine, valine, and isoleucine to account for the lack of intestinal “first-pass” metabolism [54,55,56]. This helps to avoid plasma amino acids imbalances in pediatric patients receiving PN. 

There are no data available for supporting the use of adult amino acid solutions such as Vamin^®^ (Fresenius Kabi, Bad Homburg, Germany) in infants, toddlers, children, and adolescents. As a matter of fact, even for those designed for neonatology according to the value of amino acid solutions, there is no worry regarding their use in infants/toddlers, children, and adolescents. The literature reports valuable data supporting the safety and efficiency of these pediatric amino acid mixtures in the short as well as long term (Table 1). Moreover, Vaminolact supplies taurine, an amino acid that may be conditionally essential in pediatric patients requiring long-term PN [68,116]. Taurine deficiency during the neonatal period may result in retinal dysfunction [117], as well as long-term neurodevelopmental impairment [118]. Additionally, available evidence suggests that adequate taurine provision may prevent cholestasis in newborn infants [117,119]. According to ESPGHAN/ESPEN/ESPR guidelines [50], taurine should be part of amino acid solutions for administration to the pediatric age group.

Nutritional efficacy and tolerance of Vaminolact have been proven by extensive clinical experience since its introduction to the market in 1989, demonstrated by clinical studies showing adequate weight gain, positive or improved nitrogen balance, and normal development in pediatric patients receiving PN with Vaminolact in doses in line with ESPGHAN/ESPEN/ESPR guidelines (Table 3) [50].

In 1989, Puntis et al. reported results from the first published study comparing Vaminolact in parenterally fed surgical newborn infants and infants/toddlers to a conventional adult amino acid solution based on the amino acid pattern of egg protein (Vamin) [126]. Vaminolact was initiated at 0.5 g amino acid/kg BW/day and advanced to a maximum of 2.5 g amino acid/kg/day during the 6-day study period. Plasma concentrations of all amino acids except threonine, lysine, histidine, and cysteine/cystine were closer to the reference plasma amino acid pattern of breast-fed infants than with the adult amino acid solution. In particular, infants receiving Vaminolact had a reduced risk of developing hyperphenylalaninemia and hypotyrosinemia, which have been associated with neurotoxicity in parenterally fed newborn infants [127]. From this short-term study, no significant differences in growth or nitrogen retention were seen between the two groups and no hematological or biochemical measurements changed significantly in either group. The authors concluded that Vaminolact would be likely to lessen the risk of neurotoxicity resulting from amino acid imbalance [126].

In a subsequent study comparing these two amino acid solutions (Vaminolact vs. Vamin) in critically ill newborn infants, Thornton et al. found that taurine levels recovered more rapidly with Vaminolact (n.s.), while phenylalanine levels were significantly lower at day 1 and day 3. Moreover, the administration of Vaminolact at a mean dose of 2.3 g amino acids/kg BW/day from day 3 for a mean total duration of 12 days also resulted in substantially improved nitrogen balance when compared to the adult amino acid solution [125].

A clinical study involving hospitalized infants recovering from severe malnutrition receiving Vaminolact as part of total PN showed a body weight gain after 28 days of 110 ± 5% of optimal weight gain for age. In this study, the mean non-protein energy intake from PN was 104.3 ± 8.0 kcal/kg BW/d with a mean PNDI of 210 ± 20%. Authors concluded that a non-protein energy intake twice the resting energy expenditure was optimal to achieve the targeted body weight gain in this population of malnourished infants on TPN [14].

A retrospective study by Abi Nader [13] investigated parameters of growth and nutritional status among infants/toddlers and children on HPN receiving Vaminolact or a similar amino acid solution adapted to the specific requirements of the infants (Primene) at a mean dose of 2.9 g amino acids/kg BW/day for a mean duration of 1.9 years [14]. Growth velocity for both body weight gain and size was within the normal range.

Nowadays, one should consider these pediatric amino acid solutions as more adapted than any adult amino acid solution for short- as well as for long-term use from birth to adolescence. They achieve normal body weight gain and size growth without any reported metabolic disorders.

## 5. The Role of Commercial Multi-Chamber Bags in Pediatric PN

PN formulations for pediatric patients can either be individualized, tailored to meet the specific requirements of the patient, or standardized, which may be suitable for a broad range of patients [128,129]. Individualized PN formulations are prescribed and prepared, usually on a daily basis, in the hospital pharmacy or in specialized compounding centers [129]. Individualized PN has long been regarded as the standard for PN in term infants, infants/toddlers, children, and adolescents. Nowadays there are concerns about hospital-compounded bags regarding the adequacy of nutrient delivery, as well as prescribing and compounding errors [130]. Individualizing PN in pediatric patients entails a large number of calculations, and numerous patient-specific characteristics must be accounted for. It is, therefore, particularly prone to errors, in particular concentration errors, with a reported error rate of 6% in a neonatal/pediatric ICU [131]. It is, moreover, unlikely that an expert in pediatric nutrition is readily available in each center [132].

With standardized PN formulations, in particular with ready-to-use, commercially manufactured multi-chamber bags (MCBs), this problem may be overcome since an expert nutrition care team can order the most common and suitable bags in advance [132]. Commercial MCBs can reduce infection risk, provide an adequate supply of nutrients, support adequate growth, provide ease of use, decrease prescription errors, and potentially reduce costs compared to individually compounded bags [133,134]. Thus, they can provide important benefits in pediatric patients, particularly in smaller centers where automated compounding facilities are not available. A major advantage of standardized PN bags vs. individualized PN is related to their immediate availability on demand due to easy storage and longer shelf life of approximately 2 years, while individualized formulations cannot be prepared at any time and require highly aseptic conditions, specifically trained staff, and a longer lead time for provision [135,136]. Indeed, roles for commercial MCBs have been proposed for emergency situations that make it impossible to acquire supplies, such as power failures interrupting the cooling chain or travel, or shortages of single PN components [137,138].

According to current ESPGHAN/ESPEN/ESPR guidelines [128], standard PN solutions should generally be chosen over individualized PN solutions for the majority of pediatric and newborn patients [128]. Yet, although a wide variety of commercial MCBs are available for adults, these are not optimal for infants and children as their metabolic demands and nutritional needs differ significantly, depending on age, weight, underlying disease, nutritional and hydration status, and environmental factors [3,129,139]. Accordingly, standard PN formulations designed specifically to meet the varying nutritional requirements of newborn infants, infants/toddlers, children, and adolescents have been developed and made commercially available [129]. These modern commercial MCBs contain fixed amounts of nutrients conforming to current guidelines, thus enabling an improved compliance with guidelines [128,136]. These MCBs should contain last-generation ILEs and amino acid solutions adapted for pediatric use. Vitamins must be added to the bag or delivered orally.

However, there seem to be pediatric patients whose requirements cannot be met by standard regimes, especially among patients requiring PN for longer periods. As described in the current literature, only a few children receive standard formulas in the HPN setting. According to a large national survey from France, only 0.3 to 7.2% of HPN patients received standardized formulations between 2014 and 2019, mostly during holidays or during the period of the process of weaning from PN [9]. A European survey of 61 IF teams from 20 countries found that HPN was provided in the form of individual age- and weight-specific customized bags in 78% of the IF teams, as commercial MCBs without adaptations in 25% of the teams, and as commercial MCBs customized by the pharmacy in 31% of the teams (multiple answers per team possible) [140]. Indeed, customizing commercial MCBs by individual additions of electrolytes when required may represent a promising strategy to increase the number of HPN patients that could benefit from standardized PN formulations.

## 6. Summary and Conclusions

With the evolution of practices and the extended use of PN in hospitalized as well as HPN pediatric patients, MCBs adapted to this age group are needed. The safety and efficacy of lipid and amino acid components are well documented from many reported studies, most of which are reviewed in this article. The onset of composite FO-based ILEs (SMOFlipid) calls for the development of MCBs containing these ILEs in combination with amino acid solutions adapted for use in pediatric patients. Overall, the available evidence clearly supports the benefits of SMOFlipid administered at doses in line with ESPGHAN/ESPEN/ESPR guidelines [23] in terms of improving RBC fatty acid profiles without an increased risk of EFAD, and reducing or reversing IFALD while ensuring adequate growth both in hospitalized and HPN pediatric patients (see Table 1). The advantages of MCBs have been particularly underlined for hospitalized pediatric patients, while open discussion should be promoted for their use for HPN pediatric patients.

## Figures and Tables

**Table 1:** Parenteral nutrition with SMOFlipid in newborn infants, infants/toddlers, children, and adolescents.

Study	Design Number of Patients (N)	Patients Age at Inclusion	Intervention	Control	PN Lipid Dose (g/kg BW/day)	In Line with ESPGHAN	PN Duration	Main Outcomes and Conclusions
Haines et al., 2023 [82]	Retrospective	Hospitalized infants and toddlers/children, median (IQR) age: 3.0 (0.9–11.0) years	SMOF (n = 478)	SO (n = 206)	n.a.	n.a.	SO: 9.0 (4, 25) days, SMOF: 9.0 (4, 19) days	SMOF vs. SO: Overall - Shorter LOS by 3.8 days (*p* < 0.001). - Lower risk of urinary tract infection (UTI) *p* < 0.001). ICU sub-cohort - Shorter LOS by 13.1 days *p* < 0.001). - Lower risk of UTI (*p* < 0.001).
Belza et al., 2023 [83]	Retrospective	Infants and toddlers, median age (IQR) age 4.1 (2.4–9.6) years	SMOF (n = 26)	n.a.	1.7	☑	1367 days	Normal T:T ratio in all patients. ARA levels: low in 19.2%, normal in 76.9%, and high in 3.8% of patients. DHA: normal in 7.7%, high in 92.3%; EPA: high in 100%. None with EFAD.
Hudson et al., 2023 [84]	Retrospective Multicenter	Infants Median age 5.5 weeks	SMOF (n = 35)	SO (n = 15)	SMOF: 1.8 SO: 1.5	☑	SMOF: 10.1 weeks SO: 7.66 weeks	SMOF vs. SO: Higher median serum CBil level at baseline: (*p* < 0.001). Differences resolved after 6 weeks. Proportion of patients with IFALD decreased from 54% to 20% for SMOF while stable in SO group.
Huff 2023 [85]	RCT	Surgical term newborns Median age 2–5 days	SMOF (n = 12)	SO (n = 12) SO historic (n = 12)	SMOF: 3 SO: 1	☑	Up to 12 weeks	SMOF vs. SO/SO historic: Lower weekly change in DBil levels (both *p* < 0.001). Lower DBil levels at study end (both *p* < 0.01). Higher EPA and DHA levels (*p* < 0.05), and lower ARA levels vs. control.
Yu 2023 [86]	Retrospective	Preterm and term neonates < 1 month	SMOF (n = 16)	SO (n = 136)	SMOF: median 2.7 g/kg/d SO: median 1 g/kg/d	☑	≥14 days	SMOF vs. SO: Higher incidence of cholestasis (*p* = 0.005). Shorter time to cholestasis.
Alvira-Avril 2022 [87]	Retrospective	Hospitalized and outpatient pediatric patients, newborns to adolescents < 18 years	SMOF (n = 743)	SO (n = 450)	n.a.		SMOF: 18.5 days SO: 17.5 days	SMOF vs. SO: Lower total rate of infection (*p* < 0.01). SMOF was independently associated with reduced catheter-related bloodstream infections (*p* < 0.05).
Navaratnarajah et al., 2022 [88]	Retrospective chart review N = 160	Hospitalized infants/toddlers on prolonged PN < 1 year	SMOF (n = 88)	SO (n = 72)	SMOF: 2.5 [1.7–2.8] SO 2.5 [1.9–2.8]	☑	≥28 days	SMOF vs. SO: Lower incidence of cholestasis during the study period. Lower log-transformed CBil at the end of the ILE administration (*p* < 0.02).
Goulet et al., 2022 [15]	Prospective cross-sectional N = 70	Children on HPN SMOF: 5.9 [4.1–8.4] years Weaned: 6.9 [4.0–8.7] years	SMOF (n = 46)	Weaned off PN (n = 24)	SMOF: 1.9 [1.4–2.0]	☑	2.4 ± 0.9 years	SMOF vs. weaned: Higher CBil (*p* < 0.0001) and liver enzymes (all *p* < 0.01). Higher EPA and DHA but lower MA and ARA in RBC (all *p* < 0.0001).
Goulet et al., 2021 [9]	Retrospective cross-sectional Multicenter N = 268 in 2014 N = 385 in 2019	Children on HPN Median age per year over the 5-year observation period: 62.5–84.1 months	HPN with different types of ILEs	-	1.02–1.5	☑	5 years	2014 vs. 2019: Use of a SMOF increased from 67.4% to 88.3% (*p* < 0.001). CRBSIs decreased (*p* < 0.001). Prevalence of cholestasis (CBil ≥ 20 μmol/L) low and stable during the study period.
Rumore et al., 2021 [89]	Retrospective N = 92	Infants/toddlers and children on HPN Range 3–223 months (median 11 months)	SMOF (n = 79) Switch to SMOF (n = 13)	Non-SMOF (SO or OO/SO) (n = 19)	n.a.	-	n.a.	SMOF vs. non-SMOF: Lower overall transplantation rate (*p* < 0.05). Lower mortality rate (n.s.). Higher vitamin E level and vitamin E:lipid ratio, both *p* < 0.001. Higher vitamin D level (*p* < 0.001).
Wassef et al., 2021 [90]	Prospective observational N = 16	Infants/toddlers, children and adolescents with IF	SMOF (n = 16)	-	Mean dose at initiation: 1.5 [1–2.5] Mean dose at the end: 1.6 [1–2.5]	☑	16.4 [4–33] months	End of study vs. baseline: Decrease in mean TBil (*p* < 0.05) normalization of DBil in all patients. Change in mean TBil after 4–5 months (*p* < 0.01; stable thereafter). No new cases of IFALD. No EFAD.
Daniel et al., 2021 [91]	Retrospective N = 101	Hospitalized newborn infants (preterm/term), infants/toddlers, children and adolescents SMOF: 300 [0–1095] days SO: 31 [0–795] days	SMOF (n = 60)	SO (n = 41)	Initiated: 0.5–1.0 Increased up to 2.0–3.0	☑	SMOF: 28.5 [20.75, 44] days SO: 32 [23, 55] days	SMOF vs. SO: Lower incidence of IFALD *p* < 0.05). Subgroup of patients with IFALD (n = 20), over 6 months: Lower bilirubin (*p* < 0.05).
Lezo et al., 2020 [92]	Prospective observational Multicenter N = 38	Infants/toddlers, children and adolescents on HPN SMOF; 3.3 [0.9–16.9] years OO/SO: 8.4 [1.6–18.6] years	SMOF (n = 23)	OO/SO (n = 15)	SMOF: 1.3 [0.5–2.5] OO/SO: 1.3 [0.5–1.7]	☑	SMOF: 22.2 [9.8–202] months OO/SO: 21.1 [7.0–104.0] months	SMOF vs. OO/SO and vs. healthy reference range: - Higher EPA and DHA and lower MA in plasma and RBC (all *p* < 0.01). - Lower ARA in plasma (*p* < 0.01). With both ILEs vs. healthy reference range: Lower ARA in RBC (both *p* < 0.01). With both ILEs vs. healthy reference range: no EFAD with both ILEs. Absence of liver fibrosis with both ILEs. SMOF vs. OO/SO: Median Z-score of weight, height or BMI NSD.
Nagelkerke et al., 2020 [93]	Prospective cross-sectional N = 32	Infants/toddlers, children and adolescents on HPN Median 8.0 (range 0.3–17.8) years	SMOF (n = 23) or OO/SO (n = 6)	-	Median 1.0 (range 0–2.6)	☑	Median 45 months	Varying, but substantial proportion of subjects with fibrosis in the cohort: - Significant fibrosis n = 6 and 10, respectively. - Fibrosis: n = 12 - Moderate fibrosis: n = 17.
Ho 2020 [94]	Retrospective N = 20	Children on HPN Median 6.2 years	SMOF	Pre-SMOF (SO or SO/FO combination)	2.0 [1.6–2.0]	☑	1.5 years	From SMOF initiation to 1.5 years post SMOF initiation: Increases in ARA, LA, DHA and ALA, all *p* < 0.01. Decreases in ALT and GGT; both *p* < 0.005. Slight increases in BMI z-score; n.s.
Huff 2020 [95]	Retrospective N = 47	Newborn infants, infants/toddlers, and children with IF and cholestasis (n = 42) 45 [4–1623] days	SMOF	-	Median 2.1 (range 0.8–3.0)	☑	Median 53 days (range 1–432 days)	Cholestatic patients had resolution with SMOF: 17% improved, 45% showed no response. Biochemical signs of EFAD observed in 15/28 patients with measurements available; EFAD was mild in all patients without clinical symptoms of EFAD.
Hanindita 2019 [96], 2020 [97]	RCT N = 14	Newborn infants post surgery SMOF: 14.1 ± 17.1 days MCT/SO: 14.0 ± 12.1 days	SMOF (n not reported)	SO/MCT (n not reported)	1.0–4.0	☑	SMOF: 29 ± 34 days MCT/SO: 30.0 ± 20.3 (18 ± 15 days)	SMOF vs. SO/MCT group: Decrease in SMOF vs. increase in serum IL-6 from baseline to POD 3. Significant differences in IL-6 levels before surgery (*p* = 0.048), on POD 3 (*p* = 0.013), and in changes within 3 days (*p* = 0.003).
Casson et al., 2019 [98]	Retrospective Two centers N = 44	Newborn infants and infants/toddlers during the first 8 weeks of intestinal rehabilitation SMOF: mean 7 (range 4–50) days SO: mean 8 (range 4–47) days	SMOF (n = 21)	SO (n = 23)	SMOF:wk1: 3.0 [2.0–3.0] wk4: 3.0 [1.3–3.0] wk8: 2.0 [1.5–3.0] SO:wk1: 2.0 [1.5–3.0] wk 4: 2.0 [1.5–3.0] wk 8: 1.5 [1.0–3.4]	☑	>8 weeks	SMOF vs. SO: CBil levels normalized more quickly (*p* = 0.04). Subset of infants without any EN tolerance: Lower incidence of cholestasis (78% vs. 92%, *p* = 0.057).
Belza 2019 [99]	Retrospective N = 37	Infants/toddlers with IF <12 months	SMOF (n = 17)	SO (n = 20)	2.0–3.0	☑	SMOF: 421 [203–822] days SO: 213 [104–364] days	SMOF vs. SO: Lower likelihood to reach a serum CBil of 34 µmol/L or 50 µmol/L; both *p* ≤ 0.05). With SMOF: no need for Omegaven to resolve IFALD ( *p* = 0.014). Lower median CBil 3 months after PN initiation (*p* = 0.023). Improved weight z-scores at 3 and 6 months (both *p* < 0.05). Normal range of growth in both groups.
Jiang 2019 [100]	RCT N = 160	Newborn infants after GI surgery Mean 4–5 days	SMOF (n = 74)	SO/MCT (n = 86)	1.0–3.0	☑	>2 weeks (22/24 patients > 4 weeks)	SMOF vs. SO/MCT: Infants who received lipids for >4 weeks: Lower ALT, AST and DBil levels at end of week 4 (all *p* < 0.05). NSD in weight gain or nutrition indices at end of weeks 2 and 4 months.
Lam 2018 [101]	Retrospective N = 40	Hospitalized newborn and infants/toddlers/children SMOF: 0.6 (0.1–28) mths SO: 0.8 (0.1–33) months	SMOF (n = 20)	SO (n = 20)	SMOF: 2.2 (1.8–2.5] SO: 2.1 [1.6–2.3]	☑	SMOF: 9 [5–13] weeks SO: 6 [4–13] weeks	SMOF vs. SO: Lower trajectory of CBil (*p* < 0.001) (both *p* < 0.001). Growth/nutritional status NSD.
Olszewska 2018 [102]	Prospective observational N = 17	Infants, children, and adolescents with ultra-SBS Range 0.8–14.2 years	SMOF (n = 10) or SMOF/FO (n = 5) or SO/MCT (n = 2)	-	n.a.	-	PN duration 6.6 years [0.8–14.2]	During the 1-year observation period: (SDS) of −1.2 for body mass. None of the patients had elevated CBil levels above 34.2 µmol/L.
Pereira-da-Silva 2018 [103]	RCT N = 49	Newborn infants (term/preterm) undergoing major surgery <48 h	SMOF (n = 22)	SO/MCT (n = 27)	Median cumulative dose: SMOF: 14.7 g/kg SO/MCT: 12.5 g/kg	☑	SMOF: median 16 days SO/MCT: median 18 days	SMOF vs. SO/MCT: Similar cumulative incidence rates of CBil > 1 mg/dL or >20% of TBil between groups. Cumulative incidence of hypertriglyceridemia was lower (*p* = 0.0024). Mean serum TG lower (*p* = 0.013).
Diamond 2017 [104]	RCT Multicenter N = 24	Infants/toddlers with early IFALD SMOF: mean 6.5 weeks (range 4.3–8.7) SO: mean 5.3 weeks (range 3.5–7.2)	SMOF (n = 11)	SO (n = 13)	2.0–3.0	☑	SMOF: 8 [5.5–10.5] weeks SO: 8 [5.7–10.7] weeks	SMOF vs. SO: Lower serum CBil at trial completion (primary endpoint, *p* = 0.001). Higher likeliness to have a decrease in serum CBil to 0 µmol/L over the entire study period (*p* = 0.006). Less patients with serum CBil > 50 µmol/L at primary endpoint (*p* = 0.04). Higher GGT at trial completion (*p* = 0.04).
Pichler 2015 [105]	Controlled trial non-randomized N = 67	Newborn infants, infants/toddlers, children, and adolescents with IF 0.7 [0.01–15.1] years	Mixed ILE: SMOF or OO/SO (n = 27)	SO or SO/MCT (n = 40)	n.a.	-	2–3 times per week Duration n.a.	Lower frequency of sludge and/or gallstones (*p* = 0.05). Lower liver echogenicity (*p* = 0.003). Overall, in 7 (10%) children, sludge and/or gallstones resolved spontaneously.
De Cunto 2015 [106]	Retrospective N = 42	Newborn infants (preterm/term) undergoing GI surgery 1–82 days Matched controls (n = 21, thereof 5 receiving PN due to prematurity)	SMOF (surgical group, n = 21)		0.5–3.0	☑	Mean 40 days	Surgical infants vs. matched controls: Post-surgical infants were shorter (*p* = 0.001), lighter (*p* < 0.001), and had lower fat mass content (*p* < 0.0001) than their peers at similar corrected age. Nine infants in the surgical group and 1 in the control group had PN-associated cholestasis.
Pichler 2014 [107]	Retrospective N = 177	Hospitalized infants/toddlers, children and adolescents changing to or starting PN with a FO-ILE Median 0.6 (range 0–16) years	SMOF (n = 71)	SO/MCT (n = 56)	SMOF: 2.3 ± 0.8 SO/OO/FO: 2.2 ± 0.9	☑	SMOF: median 41 (3–311) days SO/OO/FO: median 30 (3–436) days SO: median 73 (19–154)	With SMOF (baseline vs. end of treatment): - Reduced ALT (*p* = 0.006), ALP (*p* = 0.008) and GGT (*p* = 0.01). - Hyperbilirubinemia incidence decreased from 34% to 24% (*p* < 0.05). SMOF vs. SO/MCT: - Lower ALT at end of treatment (*p* = 0.01) Weight gain FO-ILEs (*p* < 0.05).
Hoffmann 2014 [108]	Retrospective N = 30	Children with haemato-oncologic disease during CT 10.69 ± 7.11 years (mean ± SD)	SMOF (n = 15)	SO (n = 15)	SMOF: 0.9 SO: 1.0	☑	>14 days	GGT increased in the SO group (*p* < 0.05). No patients developed cholestasis. Decrease in lactate dehydrogenase levels (marker of cell damage) with SMOF vs. increase in the SO group (*p* = 0.016).
Wong 2014 [109]	Retrospective N = 208	Newborn infants and infants Age not reported	SMOF or SO/OO (n = 54)	SO (n = 154)	n.a.	-	SMOF mean 19 days and SO: 21 days	Mixed ILEs vs. SO: Lower prevalence of IFALD (17% vs. 21%; *p* = 0.315)
Ariyawangso 2014 [110]	RCT N = 42	Surgical newborn infants (preterm/term) Age not reported	SMOF (n = 21)	SO (n = 21)	SMOF: 2.6 ± 0.3 SO: 2.6 ± 0.2	☑	SMOF: 22.5 ± 8.5 days SO: 20.9 ± 5.5 days	SMOF vs. SO: Lower plasma TBil (*p* < 0.001)] and DBil (*p* < 0.001)]. Increase in TBil and DBil in the SO group (*p* = 0.02 and *p* < 0.001). Decrease in TBil (*p* < 0.001) and unchanged DBil with SMOF Growth NSD at day 22.
Bishay 2012 [111]	Retrospective N = 87	Infants post surgery with or without IFALD Non-IFALD: mean 19 (1–347) days IFALD: mean 45 (4–270)	SMOF or SO/OO/FO	-	n.a.	-	Non-IFALD: 48 days (28–310) IFALD: 77 (30–276)	IFALD occurred in 33% (n = 29); 61 children receiving long-term PN (70%) achieved enteral autonomy, whereas 12 (14%) required HPN.
Muhammed 2012 [112]	Retrospective N = 17	Infants/toddlers and children with PN-associated jaundice SMOF: 12–164 weeks SO: 8–64 weeks	SMOF (n = 8)	SO (n = 9)	SMOF: 0.6–3.5 SO: 2.5–3.5	☑	SMOF: 12–148 weeks SO: 8–64 weeks	After 6 months, 5 of 8 children in the SMOF and 2 of 9 children in the SO group had total resolution of jaundice. Median TBil decreased in the SMOF group and increased in the SO group (*p* = 0.02).
Goulet 2010 [48]	RCT N = 28	Infants/toddlers and children on HPN SMOF: 30.3 ± 23.9 months SO: 38.8 ± 35.5 months	SMOF (n = 15)	SO (n = 13)	SMOF: 1.4 ± 0.5 SO: 1.4 ± 0.5	☑	SMOF: 27.3 ± 0.6 days SO: 27.5 ± 0.5 days	Baseline vs. end of study: In SMOF group: TBil decreased (*p* < 0.01). In RBC and in plasma phospholipids, EPA and DHA increased; n3-/n6-FA ratio in plasma and RBC was more elevated with SMOF (both *p* < 0.01); α-tocopherol levels increased with SMOF vs. SO (*p* < 0.05).

NSD: no significant difference; ALA: alpha-linolenic acid; ALT: alanine aminotransferase; AP: alkaline phosphatase; ARA: arachidonic acid; AST: aspartate aminotransferase; BMI: body mass index; CBil: conjugated bilirubin; CRP: C-reactive protein; DBil: direct bilirubin; DHA: docosahexaenoic acid; EFAD: essential fatty acid deficiency; EPA: eicosapentaenoic acid; FA: fatty acid; FO: fish oil; GGT: gamma-glutamyl transferase; GI: gastrointestinal; HPN: home parenteral nutrition; IF: intestinal failure; IFALD: intestinal-failure-associated liver disease; ILE: intravenous lipid emulsion; LA: linoleic acid; MA: mead acid; MCT: medium-chain triglycerides; n.a.: not available; OO: olive oil; PN: parenteral nutrition; POD: post-operative day; RBC: red blood cell; RCT: randomized controlled trial; SMOF: soybean oil/medium-chain triglycerides/olive oil/fish oil; SO: soybean oil; TBil: total bilirubin, TG: triglycerides; T:T: triene:tetraene; WFL: weight for length. Data are reported as median [IQR] unless otherwise indicated.

**Table 2:** Supply of critical amino acids with Vaminolact calculated based on dosage recommendations for total amino acids according to ESPGHAN/ESPEN/ESPR guidelines [50].

Age Group	Term Newborn Infants Up to 1 Month	Infants/Toddlers 1 Month–3 Years	Children and Adolescents 3–18 Years
Recommended total amino acid dose range acc. to ESPGHAN (g/kg BW/day)	1.5–3	1–2.5	1–2
Cysteine/cysteine (mg/kg/day)	23–46	15–38	15–31
Tyrosine (mg/kg/day)	11–23	8–19	8–15
Taurine (mg/kg/day)	7–14	5–14	5–9
Arginine (mg/kg/day)	94–188	63–157	63–126
Phenylalanine (mg/kg/day)	62–124	41–103	41–83
Valine (mg/kg/day)	83–165	55–138	55–110
Isoleucine (mg/kg/day)	71–142	47–119	47–95

**Table 3:** Parenteral nutrition with Vaminolact in term newborn infants, infants/toddlers, and children.

Study	Design Number of Patients (N)	Patients Age at Inclusion	Intervention	Control	Amino Acid Dose (g/kg BW/day)	In Line with ESPGHAN *	PN Duration	Main Outcomes
Norsa et al., 2019 [120]	Retrospective Cross-sectional N = 36	Infants/toddlers and children with neonatal short bowel syndrome. Median 9 (3–73) months	PN with Vaminolact or Primene (n = 16) on long-term PN (n = 16) or intestinal transplantation (ITx) after 2.5–9 years (n = 20)	-	n.a.	-	Follow-up: median 17 (9–20) years	Long-term PN - Six off after a mean of 8 years. - All children were alive at last FU. - Eight died after ITx. - Twelve weaned off PN NSD in Z-score for height, weight and BMI.
Abi Nader et al., 2017 [14]	Retrospective N = 17	Infants with severe malnutrition on PN for 4.5 ± 2.2 months	PN with Vaminolact or Primene	-	2.9 ± 0.3	☑	1.9 ± 0.4 years	Weight gain after 28 days of PN was 110 ± 5% of optimal weight gain/ age.
Abi Nader et al., 2016 [13]	Retrospective N = 251	Infants/toddlers on HPN Age 8.4 ± 3.6 months	HPN with Vaminolact or Primene or Vintene	-	n.a.	-	Mean duration: 1.9 ± 0.4 years	19 underwent ITx; 24 children died (10%); 91 with SBS weaned off CRBSIs: 1.7/1000 days of PN and cholestasis (51 children; 20% Patients weaned off HPN: - NSD for growth.
Struijs et al., 2013 [121]	RCT N = 23	Surgical newborn infants Gln-AA: 1 [1,2,3] days Standard-AA: 2 [1,2,3] days	GLN-AA: Pediatric AA solution with glycyl-tyrosine, Ala-Gln and acetyl-cysteine, higher in arginine and taurine vs. control (n = 17)	Standard-AA: Vaminolact (n = 6)	GLN-AA: 2.1 ± 0.5 Standard-AA: 2.1 ± 0.2	☑	GLN-AA: 7.5 ± 3.1 days Standard-AA: 8.9 ± 2.1 days	Gln-AA vs. standard AA: NSD for body weight, head circumference and pre-albumin. Plasma AA-profiles were closer to normal ranges.
Ong et al., 2012 [122]	RCT Multicenter N = 164	Surgical newborn infants and infants/toddlers Ala-Gln group: 5 (1–47) days Control: 5 (1–51) days	PN + Ala-Gln (n = 82) AA solution not specified	Standard PN (n = 82) Vaminolact in 12 centers and Primene in 2 centers	1.5	☑	Control:15.0 (13.3–16.8) days Ala-Gln: 19.0 (14.6–23.4) days	PN with Ala-Gln vs. standard PN. During exclusive PN: decreased incidence of sepsis (*p* = 0.005). NSD for growth. No side-effects.
Ikram et al., 2011 [123]	RCT N = 132	Newborn infants (preterm/term) requiring PN Age < 72 h	PN + Ala-Gln (n = 132) Prepared with Vaminolact	Standard PN (n = 138) Prepared with Vaminolact	Ala-Gln 0.6 Vaminolact dose not reported	-	n.a.	Ala-Gln vs. standard NSD for sepsis, ventilation, days NICU).
Goulet et al. 2010 [48]	RCT N = 28	Infants/toddlers and children on HPN SMOF: 30.3 ± 23.9 mths SO: 38.8 ± 35.5	SMOF (n = 15) Vaminolact for children < 3 years (n = 8)	SO (n = 13) Vaminolact for children < 3 years (n = 7)	1.8	☑	SMOF: 27.3 ± 0.6 days SO: 27.5 ± 0.5 days	NSD for clinical indices, vital signs, biological safety parameters, or growth.
Guimber et al. 1999 [124]	Retrospective N = 7	Newborn infants, infants/toddlers and children with IFALD: 2.5 yrs (4 days–11.5 yrs)	PN with Vaminolact or Primene	-	1.7 ± 0.5	☑/☒ (for intakes < 1.5)	105 days (1 day-6 months)	After PN vs. before PN: Weight/height Z-score increased (*p* < 0.05).
Thornton et al. 1991 [125]	Controlled study (not randomized) N = 25	Critically ill newborn infants requiring PN 0–4 days	PN with Vaminolact (n = 15)	PN with Vamin glucose (n = 10) Without taurine	Vaminolact: 1.8 ± 0.2 at day 1 2.3 ± 0.2 from day 3 Vamin glucose: 1.9 ± 0.1 at day 1 2.3 ± 0.1 from day 3	☑	Vaminolact: 12 ± 5 days Vamin glucose: 10 ± 3 days	No abnormalities in AA concentrations. - Vaminolact: PHE levels significantly lower at day 1 and day 3 and higher nitrogen retention on day 1. 5/15 infants regained their birth weight (BW) by PN day 7 to 11. - Vamin4/10 regained their BW by day 4 to 17.
Puntis et al. 1989 [126]	RCT N = 14	Surgical newborn infants and infants/toddlers Vaminolact group: 36 days (11–84) Vamin 9 glucose group: 16 days (11–29)	Vaminolact (n = 7)	Vamin 9 glucose (n = 7) Without taurine	0.5–2.5	☑/☒ (for intakes < 1.5)	6 days	Vaminolact vs. Vamin lower PHE, TYR and total cystine/cysteine concentration (*p* = 0.0028, *p* = 0.0004, *p* < 0.05). Mean concentrations of AA except THRE, LYS, HIST, and CYST closer to the target range. NSD in growth or nitrogen retention.

AA: amino acid; Ala-Gln: L-alanyl-L-glutamine; BMI: body mass index; BUN: blood urea nitrogen; CRBSIs: catheter-related bloodstream infections; CT: computed tomography; GI: gastrointestinal; HPN: home parenteral nutrition; IF: intestinal failure; IFALD: intestinal-failure-associated liver disease; i.v.: intravenous; n.a.: not available; NPEI: non-protein energy intake; PN: parenteral nutrition; REE: resting energy expenditure; SBS: short bowel syndrome; SMOF: soybean oil/medium-chain triglycerides/olive oil/fish oil; SO: soybean oil; 2CB: 2-chamber bag. Data are reported as median (range) or median [IQR] or mean ± SD unless otherwise indicated. * Amino acid dose supplied with Vaminolact in line with ESPGHAN recommendation [50].

## Abbreviations

ALA	alpha-linolenic acid
ALT	alanine aminotransferase
ARA	arachidonic acid
AST	aspartate aminotransferase
BW	body weight
CBil	conjugated bilirubin
DBil	direct bilirubin
DHA	docosahexaenoic acid
EFA	essential fatty acid
EFAD	essential fatty acid deficiency
EPA	eicosapentaenoic acid
FO	fish oil
GGT	gamma-glutamyl transferase
HPN	home parenteral nutrition
IF	intestinal failure
IFALD	intestinal-failure-associated liver disease
ILE	intravenous lipid emulsion
LCPUFA	long-chain polyunsaturated fatty acid
LNA	linolenic acid
MCB	multi-chamber bags
MCT	medium-chain triglyceride
OO	olive oil
PN	parenteral nutrition
PNDI	parenteral nutrition dependency index
PUFA	polyunsaturated fatty acid
RBC	red blood cell
RDA	recommended dietary allowance
SO	soybean oil
TBil	total bilirubin

## References

[1] Worthington P., Balint J., Bechtold M., Bingham A., Chan L.-N., Durfee S., Jevenn A.K., Malone A., Mascarenhas M., Robinson D.T. (2017). When Is Parenteral Nutrition Appropriate?. J. Parenter. Enter. Nutr..

[2] Koletzko B., Goulet O., Hunt J., Krohn K., Shamir R. (2005). 1. Guidelines on Paediatric Parenteral Nutrition of the European Society of Paediatric Gastroenterology, Hepatology and Nutrition (ESPGHAN) and the European Society for Clinical Nutrition and Metabolism (ESPEN), Supported by the European Society of Paediatric Research (ESPR). J. Pediatr. Gastroenterol. Nutr..

[3] Joosten K., Embleton N., Yan W., Senterre T. (2018). ESPGHAN/ESPEN/ESPR guidelines on pediatric parenteral nutrition: Energy. Clin. Nutr..

[4] Ayers P., Boullata J., Sacks G. (2018). Parenteral nutrition safety: The story continues. Nutr. Clin. Pract..

[5] Itzhaki M.H., Singer P. (2020). Advances in Medical Nutrition Therapy: Parenteral Nutrition. Nutrients.

[6] Keady S., Morgan C., Ozzard A., Chauhan B. (2010). Effect of a neonatal standard aqueous parenteral nutrition formulation on aseptic unit capacity planning. E Spen. Eur. E J. Clin. Nutr. Metab..

[7] Goulet O., Ruemmele F. (2006). Causes and management of intestinal failure in children. Gastroenterology.

[8] Vlug L.E., Nagelkerke S.C.J., Jonkers-Schuitema C.F., Rings E., Tabbers M.M. (2020). The Role of a Nutrition Support Team in the Management of Intestinal Failure Patients. Nutrients.

[9] Goulet O., Breton A., Coste M.E., Dubern B., Ecochard-Dugelay E., Guimber D., Loras-Duclaux I., Abi Nader E., Marinier E., Peretti N. (2021). Pediatric Home Parenteral Nutrition in France: A six years national survey. Clin. Nutr..

[10] Elia M. (1995). Changing concepts of nutrient requirements in disease: Implications for artificial nutritional support. Lancet.

[11] Schofield W.N. (1985). Predicting basal metabolic rate, new standards and review of previous work. Hum. Nutr. Clin. Nutr..

[12] Goulet O., Abi Nader E., Pigneur B., Lambe C. (2019). Short Bowel Syndrome as the Leading Cause of Intestinal Failure in Early Life: Some Insights into the Management. Pediatr. Gastroenterol. Hepatol. Nutr..

[13] Abi Nader E., Lambe C., Talbotec C., Pigneur B., Lacaille F., Garnier-Lengliné H., Petit L.M., Poisson C., Rocha A., Corriol O. (2016). Outcome of home parenteral nutrition in 251 children over a 14-y period: Report of a single center. Am. J. Clin. Nutr..

[14] Abi Nader E., Lambe C., Talbotec C., Dong L., Pigneur B., Goulet O. (2018). A new concept to achieve optimal weight gain in malnourished infants on total parenteral nutrition. J. Parenter. Enter. Nutr..

[15] Goulet O., Lamazière A., Abi Nader E., Talbotec C., Wolf C., Lambe C. (2022). Erythrocyte fatty acid membrane composition in children on long-term parenteral nutrition enriched with ω-3 fatty acids. Am. J. Clin. Nutr..

[16] Mehta N.M., Compher C. (2009). ASPEN Clinical Guidelines: Nutrition support of the critically ill child. J. Parenter. Enteral Nutr..

[17] Mesotten D., Joosten K., van Kempen A., Verbruggen S. (2018). ESPGHAN/ESPEN/ESPR guidelines on pediatric parenteral nutrition: Carbohydrates. Clin. Nutr..

[18] Bresson J.L., Narcy P., Putet G., Ricour C., Sachs C., Rey J. (1989). Energy substrate utilization in infants receiving total parenteral nutrition with different glucose to fat ratios. Pediatr. Res..

[19] Salas J., Girardet J.P., De Potter S., Martí-Henneberg C., Goulet O., Ricour C. (1991). Glucose versus glucose-fat mixture in the course of total parenteral nutrition: Effects on substrate utilisation and energy metabolism in malnourished children. Clin. Nutr..

[20] Preissig C.M., Rigby M.R. (2009). Hyperglycaemia results from beta-cell dysfunction in critically ill children with respiratory and cardiovascular failure: A prospective observational study. Crit. Care.

[21] Hirshberg E., Larsen G., Van Duker H. (2008). Alterations in glucose homeostasis in the pediatric intensive care unit: Hyperglycemia and glucose variability are associated with increased mortality and morbidity. Pediatr. Crit. Care Med..

[22] Dasarathy S., Dodig M., Muc S.M., Kalhan S.C., McCullough A.J. (2004). Skeletal muscle atrophy is associated with an increased expression of myostatin and impaired satellite cell function in the portacaval anastamosis rat. Am. J. Physiol. Gastrointest. Liver Physiol..

[23] Lapillonne A., Fidler Mis N., Goulet O., van den Akker C.H.P., Wu J., Koletzko B. (2018). ESPGHAN/ESPEN/ESPR/CSPEN guidelines on pediatric parenteral nutrition: Lipids. Clin. Nutr..

[24] Goulet O.J., Cai W., Seo J.M. (2020). Lipid Emulsion Use in Pediatric Patients Requiring Long-Term Parenteral Nutrition. J. Parenter. Enter. Nutr..

[25] Martindale R.G., Berlana D., Boullata J.I., Cai W., Calder P.C., Deshpande G.H., Evans D., Garcia-de-Lorenzo A., Goulet O.J., Li A. (2020). Summary of Proceedings and Expert Consensus Statements From the International Summit “Lipids in Parenteral Nutrition”. J. Parenter. Enter. Nutr..

[26] Salas J.S., Dozio E., Goulet O.J., Marti-Henneberg C., Moukarzel E., Ricour C. (1991). Energy expenditure and substrate utilization in the course of renutrition of malnourished children. J. Parenter. Enter. Nutr..

[27] Calder P.C., Waitzberg D.L., Klek S., Martindale R.G. (2020). Lipids in Parenteral Nutrition: Biological Aspects. J. Parenter. Enter. Nutr..

[28] Klek S. (2016). Omega-3 Fatty Acids in Modern Parenteral Nutrition: A Review of the Current Evidence. J. Clin. Med..

[29] Serhan C.N., Levy B.D. (2018). Resolvins in inflammation: Emergence of the pro-resolving superfamily of mediators. J. Clin. Investig..

[30] Wanten G.J., Calder P.C. (2007). Immune modulation by parenteral lipid emulsions. Am. J. Clin. Nutr..

[31] Lien E.L., Richard C., Hoffman D.R. (2018). DHA and ARA addition to infant formula: Current status and future research directions. Prostaglandins Leukot. Essent. Fat. Acids.

[32] Hoffman D.R., Boettcher J.A., Diersen-Schade D.A. (2009). Toward optimizing vision and cognition in term infants by dietary docosahexaenoic and arachidonic acid supplementation: A review of randomized controlled trials. Prostaglandins Leukot. Essent. Fat. Acids.

[33] Qawasmi A., Landeros-Weisenberger A., Bloch M.H. (2013). Meta-analysis of LCPUFA supplementation of infant formula and visual acuity. Pediatrics.

[34] Goulet O., De Potter S., Antebi H., Driss F., Colomb V., Bereziat G., Alcindor L.G., Corriol O., Le Brun A., Dutot G. (1999). Long-term efficacy and safety of a new olive oil-based intravenous fat emulsion in pediatric patients: A double-blind randomized study. Am. J. Clin. Nutr..

[35] Goulet O., Joly F., Corriol O., Colomb-Jung V. (2009). Some new insights in intestinal failure-associated liver disease. Curr. Opin. Organ Transplant..

[36] Lacaille F., Gupte G., Colomb V., D’Antiga L., Hartman C., Hojsak I., Kolacek S., Puntis J., Shamir R. (2015). Intestinal failure-associated liver disease: A position paper of the ESPGHAN Working Group of Intestinal Failure and Intestinal Transplantation. J. Pediatr. Gastroenterol. Nutr..

[37] Gura K.M., Duggan C.P., Collier S.B., Jennings R.W., Folkman J., Bistrian B.R., Puder M. (2006). Reversal of parenteral nutrition-associated liver disease in two infants with short bowel syndrome using parenteral fish oil: Implications for future management. Pediatrics.

[38] Nandivada P., Baker M.A., Mitchell P.D., O’Loughlin A.A., Potemkin A.K., Anez-Bustillos L., Carlson S.J., Dao D.T., Fell G.L., Gura K.M. (2016). Predictors of failure of fish-oil therapy for intestinal failure-associated liver disease in children. Am. J. Clin. Nutr..

[39] Hojsak I., Colomb V., Braegger C., Bronsky J., Campoy C., Domellof M., Embleton N., Fidler Mis N., Hulst J.M., Indrio F. (2016). ESPGHAN Committee on Nutrition Position Paper. Intravenous Lipid Emulsions and Risk of Hepatotoxicity in Infants and Children: A Systematic Review and Meta-analysis. J. Pediatr. Gastroenterol. Nutr..

[40] Dupont I. (1999). Peroxidation of lipid emulsions: Effects of changes in fatty acid pattern and α-tocopherol content on the sensitivity to peroxidative damage. Clin. Nutr..

[41] Goulet O., Girot R., Maier-Redelsperger M., Bougle D., Virelizier J.L., Ricour C. (1986). Hematologic disorders following prolonged use of intravenous fat emulsions in children. J. Parenter. Enter. Nutr..

[42] Clayton P.T., Bowron A., Mills K.A., Massoud A., Casteels M., Milla P.J. (1993). Phytosterolemia in children with parenteral nutrition-associated cholestatic liver disease. Gastroenterology.

[43] Hukkinen M., Mutanen A., Nissinen M., Merras-Salmio L., Gylling H., Pakarinen M.P. (2017). Parenteral Plant Sterols Accumulate in the Liver Reflecting Their Increased Serum Levels and Portal Inflammation in Children with Intestinal Failure. J. Parenter. Enter. Nutr..

[44] Pereira-Fantini P.M., Lapthorne S., Gahan C.G.M., Joyce S.A., Charles J., Fuller P.J., Bines J.E. (2017). Farnesoid X Receptor Agonist Treatment Alters Bile Acid Metabolism but Exacerbates Liver Damage in a Piglet Model of Short-Bowel Syndrome. CMGH Cell. Mol. Gastroenterol. Hepatol..

[45] Cao Y., Xiao Y., Zhou K., Yan J., Wang P., Yan W., Cai W. (2019). FXR agonist GW4064 improves liver and intestinal pathology and alters bile acid metabolism in rats undergoing small intestinal resection. Am. J. Physiol. Gastrointest. Liver Physiol..

[46] Guthrie G., Burrin D. (2021). Impact of Parenteral Lipid Emulsion Components on Cholestatic Liver Disease in Neonates. Nutrients.

[47] Bach A.C., Babayan V.K. (1982). Medium-chain triglycerides: An update. Am. J. Clin. Nutr..

[48] Goulet O., Antebi H., Wolf C., Talbotec C., Alcindor L.G., Corriol O., Lamor M., Colomb-Jung V. (2010). A new intravenous fat emulsion containing soybean oil, medium-chain triglycerides, olive oil, and fish oil: A single-center, double-blind randomized study on efficacy and safety in pediatric patients receiving home parenteral nutrition. J. Parenter. Enter. Nutr..

[49] ASPEN (2002). Guidelines for the use of parenteral and enteral nutrition in adult and paediatric patients. J. Parenter. Enter. Nutr..

[50] van Goudoever J.B., Carnielli V., Darmaun D., Sainz de Pipaon M. (2018). ESPGHAN/ESPEN/ESPR guidelines on pediatric parenteral nutrition: Amino acids. Clin. Nutr..

[51] Goulet O., DePotter S., Salas J., Robert J.J., Rongier M., Ben Hariz M., Koziet J., Desjeux J.F., Ricour C., Darmaun D. (1993). Leucine metabolism at graded amino acid intakes in children receiving parenteral nutrition. Am. J. Physiol..

[52] Dudrick S.J., Wilmore D.W., Vars H.M., Rhoads J.E. (1968). Long-term total parenteral nutrition with growth, development, and positive nitrogen balance. Surgery.

[53] Holt L.E., Snyderman S.E. (1961). The amino acid requirements of infants. JAMA.

[54] Elango R., Pencharz P.B., Ball R.O. (2002). The branched-chain amino acid requirement of parenterally fed neonatal piglets is less than the enteral requirement. J. Nutr..

[55] Kien C.L., Horswill C.A., Zipf W.B., McCoy K.S., Denne S.C. (1999). Splanchnic uptake of leucine in healthy children and in children with cystic fibrosis. Pediatr. Res..

[56] Heird W.C. (1998). Amino acids in pediatric and neonatal nutrition. Curr. Opin. Clin. Nutr. Metab. Care.

[57] Beganović N., Kok K., de Leeuw R., de Vries I.J., Schutgens R. (1983). Amino acids in parenteral nutrition of preterm infants. Comparison of oral and parenteral supply. Acta Paediatr. Scand..

[58] Andersen G.E., Bucher D., Friis-Hansen B., Nexø E., Olesen H. (1983). Plasma amino acid concentrations in newborn infants during parenteral nutrition. J. Parenter. Enter. Nutr..

[59] Bürger U., Wolf H., Fritsch U., Bauer M. (1983). Parenteral nutrition in preterm infants: Influence of respiratory treatment and effect of different amino acid compositions. J. Pediatr. Gastroenterol. Nutr..

[60] Bell E.F., Filer L.J., Wong A.P., Stegink L.D. (1983). Effects of a parenteral nutrition regimen containing dicarboxylic amino acids on plasma, erythrocyte, and urinary amino acid concentrations of young infants. Am. J. Clin. Nutr..

[61] Coran A.G., Drongowski R.A. (1987). Studies on the toxicity and efficacy of a new amino acid solution in pediatric parenteral nutrition. J. Parenter. Enter. Nutr..

[62] Sankaran K., Berscheid B., Verma V., Zakhary G., Tan L. (1985). An evaluation of total parenteral nutrition using Vamin and Aminosyn as protein base in critically ill preterm infants. J. Parenter. Enter. Nutr..

[63] Chessex P., Zebiche H., Pineault M., Lepage D., Dallaire L. (1985). Effect of amino acid composition of parenteral solutions on nitrogen retention and metabolic response in very-low-birth weight infants. J. Pediatr..

[64] Meurling S., Grotte G. (1983). Total parenteral nutrition in pediatric surgery using a new amino acid solution (Vaminolac). Acta Chir. Scand. Suppl..

[65] Pineault M., Chessex P., Lepage D., Dallaire L., Brisson G., Qureshi I. (1986). Total parenteral nutrition in very low birth weight infants with Travasol 10% blend C. J. Parenter. Enter. Nutr..

[66] Vlaardingerbroek H., Roelants J.A., Rook D., Dorst K., Schierbeek H., Vermes A., Vermeulen M.J., van Goudoever J.B., van den Akker C.H. (2014). Adaptive regulation of amino acid metabolism on early parenteral lipid and high-dose amino acid administration in VLBW infants—A randomized, controlled trial. Clin. Nutr..

[67] Vinton N.E., Laidlaw S.A., Ament M.E., Kopple J.D. (1987). Taurine concentrations in plasma, blood cells, and urine of children undergoing long-term total parenteral nutrition. Pediatr. Res..

[68] Laidlaw S.A., Kopple J.D. (1987). Newer concepts of the indispensable amino acids. Am. J. Clin. Nutr..

[69] Fitzgerald K.A., MacKay M.W. (1986). Calcium and phosphate solubility in neonatal parenteral nutrient solutions containing TrophAmine. Am. J. Hosp. Pharm..

[70] Fitzgerald K.A., MacKay M.W. (1987). Calcium and phosphate solubility in neonatal parenteral nutrient solutions containing Aminosyn PF. Am. J. Hosp. Pharm..

[71] Koletzko B., Rodriguez-Palmero M., Demmelmair H., Fidler N., Jensen R., Sauerwald T. (2001). Physiological aspects of human milk lipids. Early Hum. Dev..

[72] Gil A., Ramirez M., Gil M. (2003). Role of long-chain polyunsaturated fatty acids in infant nutrition. Eur. J. Clin. Nutr..

[73] Oliveira O.R., Santana M.G., Santos F.S., Conceição F.D., Sardinha F.L., Veiga G.V., Tavares do Carmo M.G. (2012). Composition of fatty acids in the maternal and umbilical cord plasma of adolescent and adult mothers: Relationship with anthropometric parameters of newborn. Lipids Health Dis..

[74] Agostoni C., Galli C., Riva E., Risé P., Colombo C., Giovannini M., Marangoni F. (2011). Whole blood fatty acid composition at birth: From the maternal compartment to the infant. Clin. Nutr..

[75] Koletzko B. (2016). Human Milk Lipids. Ann. Nutr. Metab..

[76] Tomsits E., Pataki M., Tölgyesi A., Fekete G., Rischak K., Szollár L. (2010). Safety and efficacy of a lipid emulsion containing a mixture of soybean oil, medium-chain triglycerides, olive oil, and fish oil: A randomised, double-blind clinical trial in premature infants requiring parenteral nutrition. J. Pediatr. Gastroenterol. Nutr..

[77] Skouroliakou M., Konstantinou D., Koutri K., Kakavelaki C., Stathopoulou M., Antoniadi M., Xemelidis N., Kona V., Markantonis S. (2010). A double-blind, randomized clinical trial of the effect of omega-3 fatty acids on the oxidative stress of preterm neonates fed through parenteral nutrition. Eur. J. Clin. Nutr..

[78] Skouroliakou M., Konstantinou D., Agakidis C., Kaliora A., Kalogeropoulos N., Massara P., Antoniadi M., Panagiotakos D., Karagiozoglou-Lampoudi T. (2016). Parenteral MCT/omega-3 Polyunsaturated Fatty Acid-Enriched Intravenous Fat Emulsion Is Associated with Cytokine and Fatty Acid Profiles Consistent with Attenuated Inflammatory Response in Preterm Neonates: A Randomized, Double-Blind Clinical Trial. Nutr. Clin. Pract..

[79] Rayyan M., Devlieger H., Jochum F., Allegaert K. (2012). Short-term use of parenteral nutrition with a lipid emulsion containing a mixture of soybean oil, olive oil, medium-chain triglycerides, and fish oil: A randomized, double-blind study in preterm infants. J. Parenter. EntEnter. Eral Nutr..

[80] Vlaardingerbroek H., Vermeulen M.J., Carnielli V.P., Vaz F.M., van den Akker C.H., van Goudoever J.B. (2014). Growth and fatty acid profiles of VLBW infants receiving a multicomponent lipid emulsion from birth. J. Pediatr. Gastroenterol. Nutr..

[81] Deshpande G., Simmer K., Deshmukh M., Mori T.A., Croft K.D., Kristensen J. (2014). Fish Oil (SMOFlipid) and olive oil lipid (Clinoleic) in very preterm neonates. J. Pediatr. Gastroenterol. Nutr..

[82] Haines K.L., Ohnuma T., Hornik C.D., Grisel B., Leraas H., Trujillo C.N., Krishnamoorthy V., Raghunathan K., Wischmeyer P.E. (2023). Change to Mixed-Lipid Emulsion From Soybean Oil-Based Lipid Emulsion in Pediatric Patients. JAMA Netw. Open.

[83] Belza C., Courtney-Martin G., Wong-Sterling S., Garofalo E., Silva C., Yanchis D., Avitzur Y., Wales P.W. (2023). Composite lipid emulsion use and essential fatty acid deficiency in pediatric patients with intestinal failure with high parenteral nutrition dependence: A retrospective cohort study. J. Parenter. Enter. Nutr..

[84] Hudson A.S., Tyminski N., Turner J.M., Silverman J.A. (2023). Intestinal Failure-Associated Liver Disease and Growth Pre- and Post-Transition to a Composite Lipid Emulsion. J. Pediatr. Gastroenterol. Nutr..

[85] Huff K.A., Cruse W., Vanderpool C. (2023). Lipid strategies to prevent intestinal failure-associated liver disease in neonates: A pilot trial. J. Parenter. Enter. Nutr..

[86] Yu L.J., Anez-Bustillos L., Mitchell P.D., Ko V.H., Secor J.D., Hurley A.P., Dao D.T., Fligor S.C., Cho B.S., Tsikis S.T. (2023). Incidence and development of cholestasis in surgical neonates receiving an intravenous mixed-oil lipid emulsion. J. Parenter. Enter. Nutr..

[87] Alvira-Arill G.R., Herrera O.R., Tsang C.C.S., Wang J., Peters B.M., Stultz J.S. (2022). Comparison of catheter-related bloodstream infection rates in pediatric patients receiving parenteral nutrition with soybean oil-based intravenous fat emulsion versus a mixed oil fat emulsion. Pharmacotherapy.

[88] Navaratnarajah N., Girard G., Sant’Anna G., Langlois H., Sant’Anna A.M. (2022). The impact of a lipid injectable emulsion (SMOF) on conjugated bilirubin levels in children receiving prolonged parenteral nutrition: A large single center experience. Clin. Nutr. ESPEN.

[89] Rumore S., McGrath K., Scott A., Sexton E., Wong T. (2021). Fat soluble vitamin status in children on home parenteral nutrition in a tertiary paediatric intestinal rehabilitation unit. Clin. Nutr. ESPEN.

[90] Wassef J., Lipkin E., Hardigan P., Duro D. (2021). Trends in liver profile and nutrition outcomes in children undergoing intestinal rehabilitation using a mixed lipid injectable emulsion. Nutr. Clin. Pract..

[91] Daniel S., Svoboda L., Chen J. (2021). Liver Function in Pediatric Recipients: A Comparison of Intralipid and Smoflipid. J. Pediatr. Pharmacol. Ther..

[92] Lezo A., D’Onofrio V., Puccinelli M.P., Capriati T., De Francesco A., Bo S., Massarenti P., Gandullia P., Marin M., Derevlean L. (2020). Plasma and Red Blood Cell PUFAs in Home Parenteral Nutrition Paediatric Patients-Effects of Lipid Emulsions. Nutrients.

[93] Nagelkerke S.C.J., Draijer L.G., Benninga M.A., Koot B.G.P., Tabbers M.M. (2021). The prevalence of liver fibrosis according to non-invasive tools in a pediatric home parenteral nutrition cohort. Clin. Nutr..

[94] Ho B.E., Chan S.C., Faino A.V., Mortensen M., Williamson N., Javid P.J., Horslen S.P., Wendel D. (2021). Evaluation of SMOFlipid in Pediatric Intestinal-Failure Patients and Its Effects on Essential Fatty Acid Levels. J. Parenter. Enter. Nutr..

[95] Huff K.A., Breckler F., Cruse W., Szeszycki E., Vanderpool C. (2021). Pediatric Smoflipid Therapy: Patient Response and Safety Concerns. J. Parenter. Enter. Nutr..

[96] Hanindita M.H., Widjaja N.A., Irawan R., Hidajat B. (2019). Influence of intravenous fish oil-enriched lipid emulsion on the inflammatory response in children post gastrointestinal surgery. Pak. J. Nutr..

[97] Hanindita M.H., Widjaja N.A., Irawan R., Hidayat B., Hariastawa I.A. (2020). Impact of Intravenous Omega-3-Enriched Lipid Emulsion on Liver Enzyme and Triglyceride Serum Levels of Children Undergoing Gastrointestinal Surgery. Pediatr. Gastroenterol. Hepatol. Nutr..

[98] Casson C., Nguyen V., Nayak P., Channabasappa N., Berris K., Panczuk J., Bhiladvala C., Dasgupta T., Piper H.G. (2020). A Comparison of Smoflipid^®^ and Intralipid^®^ in the Early Management of Infants with Intestinal Failure. J. Pediatr. Surg..

[99] Belza C., Wales J.C., Courtney-Martin G., de Silva N., Avitzur Y., Wales P.W. (2020). An Observational Study of Smoflipid vs Intralipid on the Evolution of Intestinal Failure-Associated Liver Disease in Infants with Intestinal Failure. J. Parenter. Enter. Nutr..

[100] Jiang W., Chen G., Zhang J., Lv X., Lu C., Chen H., Li W., Li H., Geng Q., Xu X. (2019). The effects of two mixed intravenous lipid emulsions on clinical outcomes in infants after gastrointestinal surgery: A prospective, randomized study. Pediatr. Surg. Int..

[101] Lam C.K.L., Church P.C., Haliburton B., Chambers K., Martincevic I., Vresk L., Courtney-Martin G., Bandsma R., Avitzur Y., Wales P.C. (2018). Long-term Exposure of Children to a Mixed Lipid Emulsion Is Less Hepatotoxic Than Soybean-based Lipid Emulsion. J. Pediatr. Gastroenterol. Nutr..

[102] Olszewska K., Ksiazyk J., Kozlowski D., Pajdowska M., Janusz M., Jaworski M. (2018). Nutritional therapy complications in children with ultra-short bowel syndrome include growth deficiency but not cholestasis. Acta Paediatr..

[103] Pereira-da-Silva L., Nóbrega S., Rosa M.L., Alves M., Pita A., Virella D., Papoila A.L., Serelha M., Cordeiro-Ferreira G., Koletzko B. (2017). Parenteral nutrition-associated cholestasis and triglyceridemia in surgical term and near-term neonates: A pilot randomized controlled trial of two mixed intravenous lipid emulsions. Clin. Nutr. ESPEN.

[104] Diamond I.R., Grant R.C., Pencharz P.B., de Silva N., Feldman B.M., Fitzgerald P., Sigalet D., Dicken B., Turner J., Marchand V. (2017). Preventing the Progression of Intestinal Failure-Associated Liver Disease in Infants Using a Composite Lipid Emulsion: A Pilot Randomized Controlled Trial of SMOFlipid. J. Parenter. Enter. Nutr..

[105] Pichler J., Watson T., McHugh K., Hill S. (2015). Prevalence of Gallstones Compared in Children with Different Intravenous Lipids. J. Pediatr. Gastroenterol. Nutr..

[106] De Cunto A., Paviotti G., Travan L., Bua J., Cont G., Demarini S. (2015). Impact of Surgery for Neonatal Gastrointestinal Diseases on Weight and Fat Mass. J. Pediatr..

[107] Pichler J., Simchowitz V., Macdonald S., Hill S. (2014). Comparison of liver function with two new/mixed intravenous lipid emulsions in children with intestinal failure. Eur. J. Clin. Nutr..

[108] Hoffmann K.M., Grabowski M., Rodl S., Deutschmann A., Schwantzer G., Sovinz P., Strenger V., Urban C., Muntean W., Hauer A.C. (2014). Short-term intravenous fish-oil emulsions in pediatric oncologic patients—Effect on liver parameters. Nutr. Cancer.

[109] Wong R.S., Walker K., Halliday R., Trivedi A. (2014). Influence of Soybean Oil or Non-Soybean Oil Based Lipid Emulsions on Parenteral Nutrition Associated Liver Disease in Late Preterm and Term Infants. Int. J. Child Health Nutr..

[110] Ariyawangso U., Puttilerpong C., Ratanachu-ek S., Anuntkosol M. (2014). Short-term safety and efficacy of fish-oil emulsions on the prevention of parenteral nutrition-associated liver disease in surgical neonates: A randomized controlled trial. Thai J. Pharm. Sci..

[111] Bishay M., Pichler J., Horn V., MacDonald S., Ellmer M., Eaton S., Hill S., Pierro A. (2012). Intestinal failure-associated liver disease in surgical infants requiring long-term parenteral nutrition. J. Pediatr. Surg..

[112] Muhammed R., Bremner R., Protheroe S., Johnson T., Holden C., Murphy M.S. (2012). Resolution of parenteral nutrition-associated jaundice on changing from a soybean oil emulsion to a complex mixed-lipid emulsion. J. Pediatr. Gastroenterol. Nutr..

[113] Anez-Bustillos L., Dao D.T., Fell G.L., Baker M.A., Gura K.M., Bistrian B.R., Puder M. (2018). Redefining essential fatty acids in the era of novel intravenous lipid emulsions. Clin. Nutr..

[114] WHO (2007). Protein and Amino Acid Requirements in Human Nutrition.

[115] Stapleton P.P., Charles R.P., Redmond H.P., Bouchier-Hayes D.J. (1997). Taurine and human nutrition. Clin. Nutr..

[116] Chesney R.W., Helms R.A., Christensen M., Budreau A.M., Han X., Sturman J.A. (1998). An updated view of the value of taurine in infant nutrition. Adv. Pediatr..

[117] Lourenco R., Camilo M.E. (2002). Taurine: A conditionally essential amino acid in humans? An overview in health and disease. Nutr. Hosp..

[118] Lima L., Obregon F., Cubillos S., Fazzino F., Jaimes I. (2001). Taurine as a micronutrient in development and regeneration of the central nervous system. Nutr. Neurosci..

[119] Kumpf V.J. (2006). Parenteral nutrition-associated liver disease in adult and pediatric patients. Nutr. Clin. Pract..

[120] Norsa L., Artru S., Lambe C., Talbotec C., Pigneur B., Ruemmele F., Colomb V., Capito C., Chardot C., Lacaille F. (2019). Long term outcomes of intestinal rehabilitation in children with neonatal very short bowel syndrome: Parenteral nutrition or intestinal transplantation. Clin. Nutr..

[121] Struijs M.-C., Schaible T., van Elburg R.M., Debauche C., te Beest H., Tibboel D. (2013). Efficacy and safety of a parenteral amino acid solution containing alanyl-glutamine versus standard solution in infants: A first-in-man randomized double-blind trial. Clini. Nutr..

[122] Ong E., Eaton S., Wade A., Horn V., Losty P., Curry J., Sugarman I., Klein N., Pierro A. (2012). Randomized clinical trial of glutamine-supplemented versus standard parenteral nutrition in infants with surgical gastrointestinal disease. Br. J. Surg..

[123] Mohamad Ikram I., Quah B., Noraida R., Djokomuljanto S., Faris Irfan C., Van Rostenberghe H. (2011). A randomised controlled trial of glutamine-enriched neonatal parenteral nutrition in Malaysia. Singap. Med. J..

[124] Guimber D., Michaud L., Ategbo S., Turck D., Gottrand F. (1999). Experience of parenteral nutrition for nutritional rescue in children with severe liver disease following failure of enteral nutrition. Pediatr. Transplant..

[125] Thornton L., Griffin E. (1991). Evaluation of a taurine containing amino acid solution in parenteral nutrition. Arch. Dis. Child..

[126] Puntis J., Ball P., Preece M., Green A., Brown G., Booth I. (1989). Egg and breast milk based nitrogen sources compared. Arch. Dis. Child..

[127] Puntis J.W., Edwards M.A., Green A., Morgan I., Booth I.W., Ball P.A. (1986). Hyperphenylalaninaemia in parenterally fed newborn babies. Lancet.

[128] Riskin A., Picaud J.C., Shamir R. (2018). ESPGHAN/ESPEN/ESPR guidelines on pediatric parenteral nutrition: Standard versus individualized parenteral nutrition. Clin. Nutr..

[129] Ferreira M., Guerra P., Ferreras C., Espinheira M.D.C., Trindade E., Dias J.A. (2021). Could Commercial Formulations Replace Individualized Prescription in Pediatric Home Parenteral Nutrition?. J. Pediatr. Gastroenterol. Nutr..

[130] Meyer R., Timmermann M., Schulzke S., Kiss C., Sidler M., Furlano R. (2013). Developing and implementing all-in-one standard paediatric parenteral nutrition. Nutrients.

[131] Hermanspann T., Schoberer M., Robel-Tillig E., Hartel C., Goelz R., Orlikowsky T., Eisert A. (2017). Incidence and Severity of Prescribing Errors in Parenteral Nutrition for Pediatric Inpatients at a Neonatal and Pediatric Intensive Care Unit. Front. Pediatr..

[132] Riskin A., Shiff Y., Shamir R. (2006). Parenteral nutrition in neonatology—To standardize or individualize?. Isr. Med. Assoc. J..

[133] Mena K.D.R., Espitia O.L.P., Vergara J.A.D. (2018). Management of Ready-to-Use Parenteral Nutrition in Newborns: Systematic Review. J. Parenter. Enter. Nutr..

[134] Simmer K., Rakshasbhuvankar A., Deshpande G. (2013). Standardised parenteral nutrition. Nutrients.

[135] Adamkin D.H., Radmacher P.G. (2014). Current trends and future challenges in neonatal parenteral nutrition. J. Neonatal Perinatal Med..

[136] Lapillonne A., Berleur M.P., Brasseur Y., Calvez S. (2018). Safety of parenteral nutrition in newborns: Results from a nationwide prospective cohort study. Clin. Nutr..

[137] Kumpf V.J. (2019). Challenges and Obstacles of Long-Term Home Parenteral Nutrition. Nutr. Clin. Pract..

[138] Holcombe B., Mattox T.W., Plogsted S. (2018). Drug shortages: Effect on parenteral nutrition therapy. Nutr. Clin. Pract..

[139] Hill S., Ksiazyk J., Prell C., Tabbers M. (2018). ESPGHAN/ESPEN/ESPR/CSPEN guidelines on pediatric parenteral nutrition: Home parenteral nutrition. Clin. Nutr..

[140] Neelis E., de Koning B., van Winckel M., Tabbers M., Hill S., Hulst J. (2018). Wide variation in organisation and clinical practice of paediatric intestinal failure teams: An international survey. Clin. Nutr..

